# Editorial: Insights in sleep and circadian rhythms: 2021

**DOI:** 10.3389/fnins.2023.1133907

**Published:** 2023-01-17

**Authors:** Ritchie E. Brown, Luis de Lecea

**Affiliations:** ^1^Laboratory of Neuroscience, Department of Psychiatry, VA Boston Healthcare System and Harvard Medical School, West Roxbury, MA, United States; ^2^Department of Psychiatry and Behavioral Sciences, Stanford University School of Medicine, Stanford, CA, United States; ^3^Wu Tsai Neurosciences Institute, Stanford University, Stanford, CA, United States

**Keywords:** sleep, pain, epilepsy, chronotype, coma, fatigue, psychiatric

We are pleased to introduce this Research Topic of the Sleep and Circadian Rhythms section of Frontiers in Neuroscience entitled “*Insight in Sleep and Circadian Rhythms: 2021*.” Recent years have seen increased recognition that impaired sleep and circadian rhythms can lead to a wide range of deleterious effects for the body and the brain. Conversely, our improved understanding of the basic neurobiology of these processes allows novel targeted translational approaches to improve sleep and arousal (Brown et al., 2022). This collection of articles focuses on the basic neurobiology of sleep and circadian rhythms and the application of that knowledge to coma, sleep disorders, fatigue, psychiatric disorders, pain, and epilepsy.

Although mankind has been interested in sleep and dreams throughout history, the modern study of the brain regions, neurotransmitters and mechanisms which wake us up and put us to sleep began around 70–100 years ago with the seminal studies of researchers and clinicians who analyzed the effects of transections or lesions of different parts of the brainstem. Grady et al. review these classic studies as well as more recent studies to assess our current understanding of the brainstem regions and pathways which are required for wakefulness and for consciousness. Both the older studies and more recent approaches suggest that damage to a circumscribed region of the upper brainstem produces a coma-like state. Grady et al. conclude that damage to a so-far unidentified group of neurons in this region, distinct from the well-known cholinergic and noradrenergic neurons, is responsible for coma, setting the stage for future cell-specific studies which could identify markers of these neurons and allow targeted treatments for disorders of consciousness.

Three articles in this Research Topic address the effects of disrupted sleep. Lack of adequate sleep affects virtually all physiological functions. Kim et al., review what functional magnetic resonance imaging studies have informed us about the brain regions and functional networks that mediate the effects of sleep deprivation on long-term memory and cognitive control processes in humans. Decreased responses are observed in prefrontal and parietal cortices and the hippocampus whereas thalamic and basal ganglia regions show increased responses, possibly a compensatory mechanism to maintain cortical activity in the presence of increased levels of inhibitory sleep homeostatic factors. They propose that targeted blue light therapy may be one way to partially ameliorate these deficits. Impaired cognition due to fatigue is a major cause of accidents on the road, in the workplace and in the home and leads to poor judgement and increased risk-taking behavior. Kayser et al. propose that real-time detection of fatigue using multiple sensors assessing eye-movement, temperature, and heart-rate variability could prove more effective than single measure systems and allow closed-loop systems which assess tiredness as well as the effectiveness of pharmacological or non-invasive stimulation treatments. Sleep deprivation also affects sensory perception. Amongst sensory systems, pain appears to be particularly sensitive to sleep loss, as described by Kourbanova et al.. In reviewing the human and rodent literature they conclude that loss of NREM sleep is a major driver of pain hypersensitivity produced by sleep loss. Importantly, female subjects tend to develop more pain hypersensitivity than males. Multiple levels of the pain processing hierarchy are likely to be involved, including nociceptors, spinal pathways, brainstem as well as central cortical and subcortical systems involved in emotional and cognitive components of the pain response, and descending pathways. Understanding the effect of sleep disruption on these pathways will likely improve pain management in hospital and in outpatient settings.

The timing, amount and architecture of sleep are regulated by the interaction of the time of day (circadian process C) and the amount of time spent awake (homeostatic factor, process S) (Borbely, 1982). There is wide interindividual variation in these processes. We all know people who get up early and go to bed early (so-called larks) or those who prefer to get up late and go to bed late (night owls). Putilov and Donskaya model the homeostatic process in the two extreme chronotypes based on pre-existing data, concluding that it operates similarly. Thus, changes in the circadian system are likely to account for the different chronotypes. Further support for a role of changes to the circadian system is provided by the analysis of polymorphisms in the core and accessory “clock” genes which generate ~24-h rhythms and their entrainment by light and other zeitgebers, as discussed in the article by Zou et al., who comprehensively review the relationship between different chronotypes and psychiatric disorders. Although the direction of causality remains unclear, they conclude that considerable evidence supports an association of evening chronotypes with depressive disorders, substance abuse and eating disorders, which is likely driven at least in part by sleep disruption due to “social jetlag” i.e., a mismatch between preferred sleeping time and societal timing of work or school. The propensity for an evening or morning chronotype also varies across the lifespan with teenagers tending to be night owls and a change to a morning chronotype occurring as we age. Aging is also associated with disrupted sleep, which recent studies in mice link to changes in the excitability of hypocretin neurons (Li et al., 2022).

Circadian and homeostatic factors also affect the excitability of cortical and subcortical circuits through modulation of excitatory-inhibitory balance. Alterations in excitatory-inhibitory balance are involved in a variety of important neuropsychiatric disorders. One example is epilepsy. The article by Kreitlow et al. reviews how circadian and longer duration cycles affect the propensity for sudden unexpected death in treatment-resistant epilepsy (SUDEP) and discusses the possibility to prevent these deaths through seizure-prediction technologies and time-dependent seizure management strategies.

The research described in these seven thought-provoking articles provides many exciting examples of how fundamental research into sleep and circadian rhythms has the potential to affect a broad swathe of pathological conditions.

## Author contributions

RB drafted the manuscript. LL reviewed and edited the manuscript. RB and LL organized this Research Topic. All authors contributed to the article and approved the submitted version.
